# Construction and Immunological Evaluation of CpG-Au@HBc Virus-Like Nanoparticles as a Potential Vaccine

**DOI:** 10.1186/s11671-016-1554-y

**Published:** 2016-07-19

**Authors:** Yarun Wang, Yue Wang, Ning Kang, Yongliang Liu, Wenjun Shan, Shengli Bi, Lei Ren, Guohong Zhuang

**Affiliations:** Department of Biomaterials and Fujian Collaborative Innovation Center for Exploitation and Utilization of Marine Biological Resources, College of Materials, Xiamen University, Xiamen, 361005 People’s Republic of China; Organ Transplantation Institute, Anti-Cancer Research Center, Medical College, Xiamen University, Xiamen, 361005 People’s Republic of China; National Institute for Viral Disease Control and Prevention, China Center for Disease Control and Prevention, Changbai Road 155, Changping District, Beijing, 102206 People’s Republic of China; State Key Laboratory for Physical Chemistry of Solid Surfaces, Xiamen University, Xiamen, 361005 People’s Republic of China; Fujian Collaborative Innovation Center for Exploitation and Utilization of Marine Biological Resources, Xiamen University, Xiamen, 361005 People’s Republic of China

**Keywords:** Virus-like nanoparticles, CpG ODNs, Gold nanoparticles, Immune response

## Abstract

Different types of vaccines have been developed to elicit active immunization to treat various diseases, while suffer from limitation of efficacy. Herein, a novel immunostimulatory nanocomposite (CpG-Au@HBc VLP) was rationally designed by self-assembling engineered virus-like particles encapsulating CpG-gold nanoparticle conjugates through electrostatic interactions. The monodispersed and uniformly sized CpG-Au@HBc VLP showed increased CD4^+^, CD8^+^ T cell numbers and stronger secretion of cytokine interferon-gamma than HBc VLPs adjuvanted with conventional Freund’s adjuvant. Furthermore, the use of Au nanoparticles also generated enhanced immunogenicity of CpG and VLPs on both humoral and cellular immune pathways, as followed from increased expressions of total HBc-specific antibody titer, CD4^+^ T cells, CD8^+^ T cells, cytokine interleukin-4, and interferon-gamma. These findings demonstrated that CpG-Au@HBc VLP nanocomposite could induce robust cellular and humoral immune response, which could be a potential vaccine for future prophylactic and therapeutic application.

## Background

Vaccines aim at generating the body’s protective and therapeutic immune responses against many diseases, such as infectious diseases and cancers [1]. Conventional vaccines, which are composed of attenuated live and inactivated vaccines, mostly stimulate humoral immunity, and result in safety concerns [2]. Thus, it is necessary to develop safety vaccines, which can elicit not only humoral but also cellular immunity. With the progress of immunology and molecular biology, a series of biomolecules have been utilized as antigens to construct new vaccine formulations, including recombined virus or bacteria [3], dendritic cells [4], T cells [5], nucleic acids [6, 7], and peptides [8]. Among these antigens, some of the recombined protein, derived from viruses (such as hepatitis B and C), have been reported to be able to induce host immune responses [9]. The hepatitis B core protein (HBc) virus-like particles (VLPs) are self-assembled by hepatitis B virus core proteins in recombinant expression systems (*Escherichia coli* (*E. coli*)) [10, 11]. HBc VLPs are hollow and stable without incorporating genetic materials but still keep similar conformations to the wild virus. They could be dissociated and re-assembled relying on denaturant stock solution such as urea and guanidine by breaking the disulfide bond among monomers in the VLPs [12]. Moreover, HBc VLPs have high proper immunogenicity on B cell, T cell, and cytotoxic T lymphocyte (CTL) level [9]. Thus, they are promising candidate vaccine antigens and nanocarriers to deliver genes, drugs, or other therapeutics with good biocompatibility and non-infectiousness. Considering of these features, HBc VLPs could be employed to construct new types of vaccine formulation that simultaneously induced robust humoral and cellular responses [13, 14]. However, the main concern for using HBc VLPs as vaccines is that it is insufficient enough to induce immune responses by using HBc antigens alone.

Adjuvant is responsible for stronger efficacy of vaccine and subsequently significant immune responses. Oligodeoxynucleotides (ODNs) containing unmethylated CpG motifs (CpG ODNs) have been utilized as potent adjuvants with negligible toxicity, due to their wide stimulation to macrophage cells, dendritic cells, natural killer (NK) cells, and B cells [15]. CpG ODNs can not only activate innate and acquired immune responses but also preferentially induce T helper 1(Th1) responses on antigen-specific T cell responses, which is more preferred for immunization against many diseases [16, 17]. Nevertheless, some drawbacks are still needed to conquer. CpG ODNs cannot penetrate through cell membrane and are easy to be cleared by nucleases in plasma or cytoplasm [18]. Some transfection agents have been employed to improve delivery of CpG ODNs into targeting cells [19, 20], due to their potential to prolong the tissue retention time of loaded therapeutics, and to reduce exposure time of CpG ODNs in systemic circulation. The rapid development of nanotechnology also raises new promises in the generation of novel adjuvant systems based on nanoparticles. These nanoparticles are well suited for delivery of immune therapies and can cause immune responses by themselves [21–23]. Gold nanoparticles (Au NPs), with excellent biocompatibility, easily tuned sizes and modification, have been extensively applied to antigens and immune adjuvant delivery [24]. They are able to protect immune therapeutic molecules like CpG ODNs from degradation. Some kinds of Au NPs, for example, gold nanorods (Au NRs), were also found to exhibit adjuvant activities although the detailed mechanisms still need further investigation [25]. Nevertheless, the co-immune effect of the CpG ODNs, Au NPs, and HBc VLPs still lack of research.

In the present work, we developed CpG-Au@HBc VLPs as an immunostimulatory nanocomposite in which the CpG ODNs adjuvant was conjugated with Au NPs and co-encapsulated by genetically engineered HBc VLPs. The designed nanocomposite showed the ability to induce robust specific T cell response and enhanced Th1-type immune response than HBc VLPs co-immune with conventional adjuvant. It was also revealed that Au NPs could enhance the immune stimulation of CpG ODNs on both humoral and cellular immune response. As a result, the CpG-Au@HBc VLPs might be a potential vaccine for future prophylactic and therapeutic application.

## Methods

### Materials

Chemicals including chloroauric acid trihydrate (HAuCl_4_·3H_2_O), sodium citrate, sodium chloride (NaCl), potassium chloride (KCl), sodium phosphate dibasic (Na_2_HPO_4_), potassium phosphate monobasic (KH_2_PO_4_), 10 % sodium azide (NaN_3_), Tris(hydroxymethyl)aminomethane (Tris-HCl), isopropyl-*β*-d-thiogalactoside (IPTG), Triton, ammonium sulfate ((NH_4_)_2_SO_4_), sodium dodecyl sulfate (SDS), glycerol, glycine, and Tween-20, were purchased from Sinopharm Chemical Reagent Co., Ltd (Shanghai, China). Bovine serum albumin (BSA), Freund’s adjuvant complete and Freund’s adjuvant incomplete were purchased from Sigma-Aldrich (Sigma-Aldrich St Louis, MO, USA). Horseradish peroxidase (HRP) conjugated anti-mouse antibody, 3, 3′ 5, 5′-tetramethylbenzidine (TMB), PE/Cy5 anti-mouse CD8a, and FITC-CD4 anti-mouse CD4 were purchased from Ebioscience Biotechnology (USA). High affinity purification purified CpG ODNs (5′-TCCATGACGTTCCTGACGTT-SH-3′) were synthesized by Sangon Biotech Co., Ltd (Shanghai, China). Unless otherwise noted, all chemicals were analytical grade and used without further purification. Milli-Q water (less than 18.2 MΩ cm) was used throughout the whole experiment.

### Expression and Purification of Full-Length HBc VLPs

The coding sequence of the full-length HBc antigen (HBcAg) was amplified from the plasmid pET-43.1a. Amino acid sequence of the full-length HBcAg was the same as the published report [11]. The expression and purification of full-length HBc VLPs were conducted according to our previous methods [26]. Briefly, prokaryotic vector *E. coli* BL21 (DE3) strain containing the expression plasmid was cultured and induced to express HBc VLPs. After the expression, HBc VLPs were separated and purified successively (detected by sodium dodecyl sulfate polyacrylamide gel electrophoresis) by salting out with saturated ammonium sulfate, DEAE ion-exchange chromatography, and Sepharose CL 4B molecular exclusion chromatography. The purified HBc VLPs were ultrafiltered to reach a final concentration of 2 mg/mL in PBS and stored at −20 °C.

### Synthesis of Au NPs

Citrate-stabilized gold nanoparticles with small size were prepared using a modified seed-mediated method reported by Frens et al. [27]. Briefly, HAuCl_4_ aqueous solution (250 μL, 100 mM) was added to 100 mL ultrapure water and heated to boiling, then trisodium citrate solution (3.5 mL, 1 %, *w*/*v*) was added quickly under vigorous stirring. After reaction for 20 min, the Au solution was cooled down to room temperature. The as-synthesized Au NPs were kept at 4 °C.

### Functionalization of Au NPs with CpG ODNs

The assembly of Au NPs with CpG ODNs was conducted by using a salt-aging approach as follows. Firstly, sulfhydryl-modified CpG ODNs (5 μL, 100 μM) and Au NPs solution (5 mL, 10 nM) were mixed at 16 °C for 12 h. The solution was adjusted to 0.1 % SDS and 10 mM of PBS buffer (pH 7.4). Then the concentration of NaCl in the solution was slowly increased to 0.1 M in a 6-h time window by stepwise addition of 1 M NaCl aqueous solution. The mixture solution was shaken for 12 h at room temperature to make sure the Au NPs were fully covered by thiolated CpG ODNs. CpG-Au NP conjugates were then centrifuged (18,000 rpm, 20 min, 4 °C) and washed with 0.1× PBS buffer for three times to remove the extra CpG ODNs. The final precipitate was dissolved in PBS for further use.

To quantify the accurate amount of CpG ODNs bound to each Au NP, a fluorescence-based method was used [28]. Briefly, conjugation of 5′-FAM-CpG ODNs with Au NPs was performed with the same protocol as mentioned above. 2-mercaptoethanol (final concentration 12 mM) was then added to the solution and shaken overnight to replace the binding 5′-FAM-CpG ODNs. The solutions containing displaced 5′-FAM-CpG ODNs were separated from the Au NPs by centrifugation. Then the fluorescence emission intensity of FAM molecules at 520 nm was measured to quantify the concentration of displaced CpG ODNs. Dividing by the Au NPs concentration obtained from UV-vis spectroscopy measurements [29], the number of CpG ODNs released from per Au nanoparticle was determined.

### Construction of CpG-Au@HBc and CpG@HBc VLPs

The HBc VLPs were firstly dissociated by adding dissociating buffer (3 M urea, 150 mM NaCl, and 50 mM Tris-HCl) with a final concentration of 1 mg/mL and incubated for 3 h at room temperature. Then CpG-Au NP conjugate solution (2.5 mL, 20 nM) or free CpG ODNs (10 μL, 1 mg/mL) were added and incubated for 30 min under slight shaking to form CpG-Au@HBc VLPs or CpG@HBc VLPs, respectively. Finally, samples were dialyzed overnight against reassembling buffer (10 mM Tris, 150 mM NaCl, 10 % glycerol, 1 % glycine, pH 8.0) at 4 °C, and detected by agarose gel electrophoresis.

### Mice and Immunization

Six-week-old pathogen-free female BALB/c mice were obtained from Shanghai Laboratory Animal Center of Chinese Academy of Sciences (China) and kept at the Animal Center of Xiamen University. All animal experiments were conducted according to the guidelines of the Institutional Animal Care and Use Committee. The mice were randomly divided into five groups (six mice per group). Then the mice were injected intraperitoneally with following vaccine formulations (0.2 mL) (a) 50 μg HBc VLPs only, (b) CpG@HBc VLPs: 50 μg HBc VLPs encapsulated 10 μg CpG ODNs, (c) CpG-Au@HBc VLPs: 50 μg HBc VLPs encapsulated CpG-Au NP conjugates containing 10 μg CpG ODNs, (d) CpG-Au + HBc VLPs: 50 μg HBc VLPs mixed with CpG-Au NP conjugates containing 10 μg CpG ODNs, (e) CFA/IFA + HBc VLPs: HBc VLPs mixed with Freund’s adjuvant (positive control): 50 μg HBc VLPs mixed with 50 μg complete Freund’s adjuvant (CFA) for the first injection, and 50 μg HBc VLPs mixed with 50 μg incomplete Freund’s adjuvant (IFA) for the other three injections, and (f) PBS (0.01 M, pH = 7.4) as blank control. Mice were immunized on days 0, 7, 14, and 21, respectively. After immunization for the 4 weeks, all mice were sacrificed on day 28. The sera and splenocytes of mice were harvested for further analysis.

### Detection of HBc-Specific IgG Antibody

96-well plates were coated with HBc VLPs in coating buffer (1 mg/mL in 50 mM sodium carbonate buffer, pH 9.6, 100 μL per well) overnight at 4 °C. Wells were washed three times with PBS containing 0.05 % Tween-20 then blocked by 1 % BSA at 37 °C for 1 h. After washing with PBS, diluted mouse serum samples collected after each immunization were incubated at 37 °C for 1 h, followed by washing three times, 100 μL of horseradish peroxidase (HRP)-conjugated anti-mouse antibody at different dilutions (1:100, 1:1000, and 1:10,000) were added to wells. The plates were incubated at 37 °C for 1 h and washed again. TMB substrate solution was added to each well and incubated for 15 min at 37 °C. Then H_2_SO_4_ was added to have color reaction. Finally, the absorbance of samples at 450 nm was measured with microplate reader.

### Detection of CD4^+^ and CD8^+^ T Cells in Mice Splenocytes

The splenocytes were separated after mice of all five groups were euthanized. Then, splenocytes were stained by fluorescent antibodies (PE/Cy5 anti-mouse CD8a and FITC anti-mouse CD4, respectively) on ice for 30 min, and cells were subsequently washed with ice-cold PBS, containing 0.1 % NaN_3_ and 0.5 % BSA. The expression of CD4^+^ and CD8^+^ T cells were determined by flow cytometry analysis.

### Cytokine Test

The level of cytokine interferon-gamma (IFN-*γ*), interleukin-2 (IL-2), and interleukin-4 (IL-4) from mice serum in the presence of the four different vaccine formulations were analyzed with the cytokine enzyme-linked immunosorbent assay (ELISA) kits (R&D system, USA). All operations were performed following the manufacturer’s instructions.

### Characterization

Transmission electron microscopy (TEM) images were obtained by a JEM-1400 (JEOL, Japan) operated at an accelerating voltage of 100 kV. Protein samples for TEM analysis were prepared by spreading a drop of the sample dilute dispersion on carbon film-coated copper grids followed by staining with 1 % uranyl acetate. Dynamic light scattering (DLS) and zeta potential distribution were measured on a Malvern Zetasizer Nano ZS (Malvern, UK). UV-vis absorption spectra were acquired with a DU 800 UV-vis spectrophotometer (Beckman Coulter, Fullerton, CA). The fluorescence emission spectra were obtained by FluoroMax-4 spectrometer (Jobin Yvon Horiba, France). Flow cytometry analysis was performed on a Coulter Epics XL flow cytometer (Beckman Coulter, Fullerton, CA). Optical absorbance measurements in ELISA assays were performed on Bio-Rad Model 680 microplate reader (Bio-Rad, USA).

### Statistics

Data for each experiment were presented as mean values ± standard deviation (SD). Comparisons between two groups were performed by two-tailed Student’s *t* test. *p* values below 0.05 were considered as significant and indicated with * in the figures.

## Results and Discussion

### Characterization of HBc VLPs

**Fig. 1:**
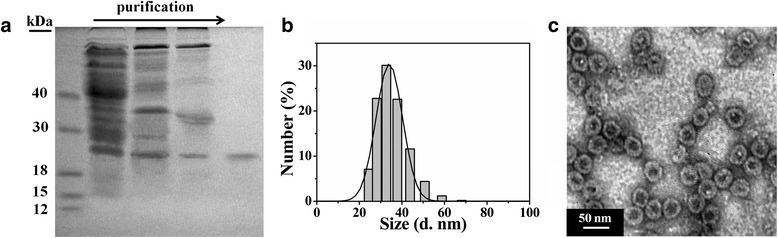
Physicochemical characterization of purified HBc VLPs. **a** SDS-PAGE analysis of HBc VLPs in different stages of purification, from left to right: marker, bacterial liquid, protein separation from salting out, ion-exchange chromatography and molecular exclusion chromatography, marker. **b** DLS data of HBc VLPs. **c** TEM analysis of negatively stained HBc VLPs

VLPs generated from HBc proteins have been constructed to inducibly and efficiently express in *E. coli* system and purified by salting out, ion-exchange chromatography, and molecular exclusion chromatography. Sodium dodecyl sulfate-polyacrylamide gel electrophoresis (SDS-PAGE) result (Fig. 1a) showed the significantly improved purity of proteins through purification by salting out and chromatography. The monomer molecular weight of full-length HBc VLPs was measured around ~20.9 kDa. The hydrodynamic size of HBc VLPs was ~34 nm by DLS analysis (Fig. 1b). The protein demonstrated good capability for self-assembling into icosahedral particles (average size was 30.8 ± 1.3 nm) with empty inner cavity of 20.6 ± 0.7 nm in diameter according to TEM negative staining investigation (Fig. 1c). The as-prepared full-length HBc VLPs showed consistent size and morphology with that reported in previous studies [13, 30].

### Characterization of CpG-Au NP Conjugates

**Fig. 2:**
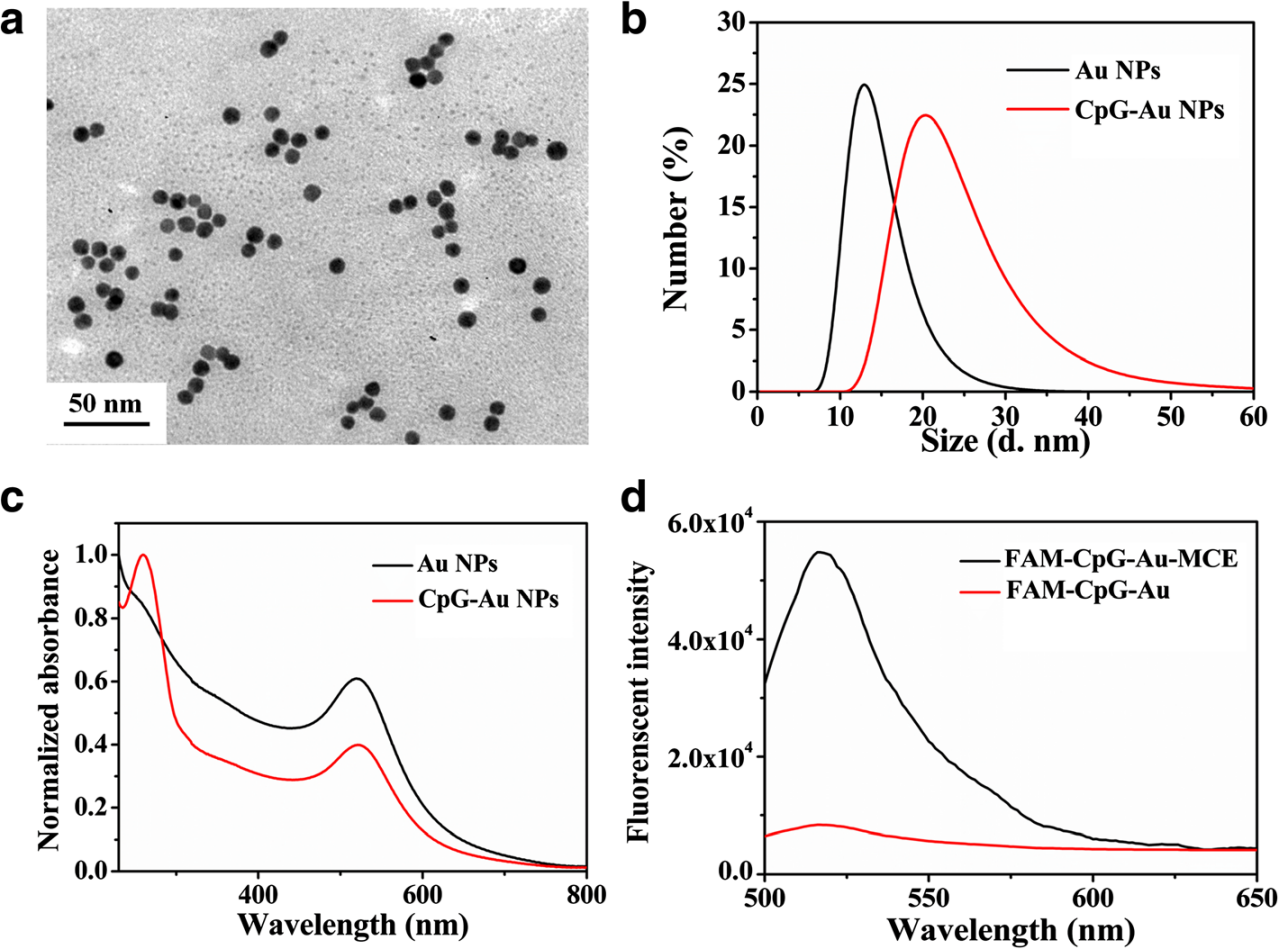
Characterization of Au NPs before and after functionalized with CpG ODNs. **a** TEM images of the as-synthesized Au NPs; **b** size distribution; and **c** UV-vis spectra of Au NPs and CpG-Au NP nanoconjugates. **d** The fluorescence spectra of the supernatant before (*red line*) and after (*black line*) displacement by mercaptoethanol (MCE) of the fluorescein-tagged, thiolated CpG ODNs

Small-sized Au NPs were prepared according to a modified seed-mediated method. The TEM image in Fig. 2a showed the obtained Au NPs were homogeneous and monodisperse. The average size of Au NPs was measured at 10.5 ± 1.2 nm. Thiolated CpG ODNs (5′-TCCATGACGTTCCTGACGTT-3′-SH), an effective sequence to stimulate immune responses of mice [31], could be successfully modified on Au NPs to form CpG-Au conjugations through Au–S bond. After CpG ODNs coating, the hydrodynamic size of Au NPs was increased from 12.4 to 20.5 nm (Fig. 2b), and an absorption peak at 280 nm was observed (Fig. 2c), corresponding to the characteristic absorption peak of CpG ODNs. These results demonstrated that thiolated CpG ODNs have been loaded on the surface of Au NPs. According to the calculation, for the as-prepared Au NPs, the surface coverage is ∼31 CpG ODNs per nanoparticle (Fig. 2d).

### Characterization of HBc VLPs Encapsulated CpG-Au NPs and CpG

**Fig. 3:**
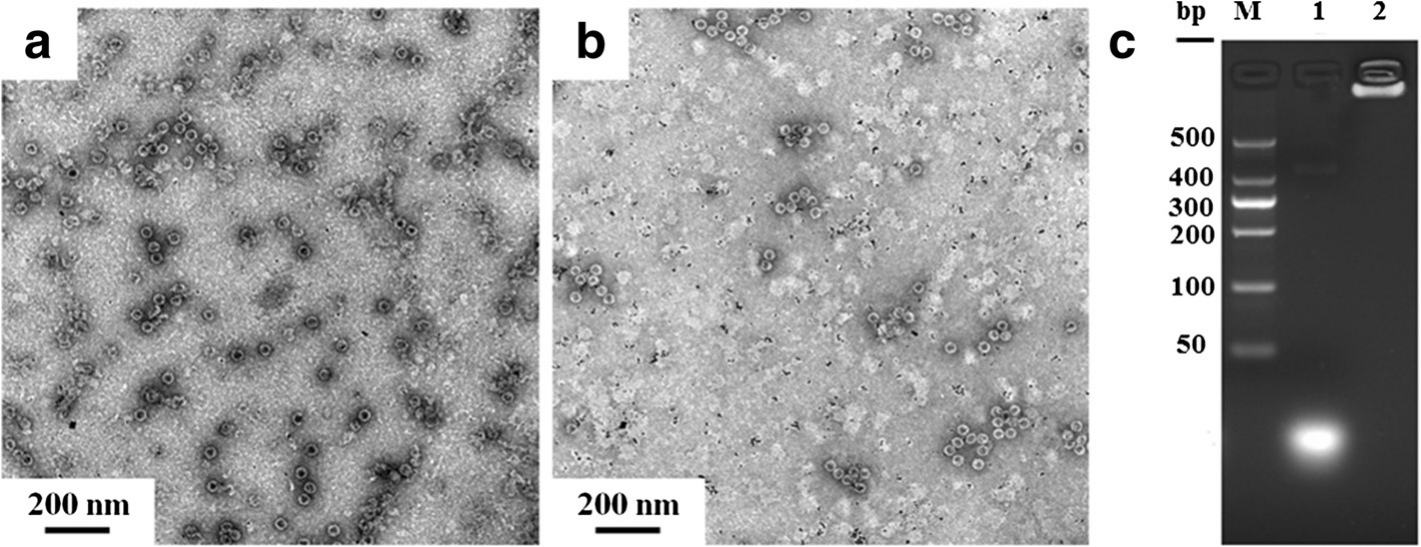
Characterization of CpG-Au@HBc VLPs and CpG@HBc VLPs. TEM images of negatively stained samples of HBc VLPs encapsulated **a** CpG-Au NPs and **b** Au NPs. **c** Agarose gel electrophore analysis of CpG ODN (20 bp) encapsulated into HBc (lane 2), lane 1: control experiment with free CpG at the same mass, lane M: marker

To reach an enhanced immunostimulatory effect, we integrated antigen with adjuvant by designing CpG-Au@HBc VLPs nanocomposites. The HBc VLPs were firstly disassembled after incubation with denaturant stock solution to expose the arginine-rich blocks of each HBc subunit. The CpG-Au conjugates were subsequently added and co-incubated for another 20 min using slight vibrational motion. The reassembly of CpG-Au core-containing VLPs were performed by dialysising the dissociated HBc VLPs subunits against an assembling buffer. The as-prepared CpG-Au@HBc VLPs were finally purified by centrifugation. TEM images (Fig. 3a) of CpG-Au@HBc VLPs indicated that Au nanoparticles with higher contrast were successfully enclosed inside the HBc VLPs. Moreover, the obtained CpG-Au@HBc VLPs showed monodisperse, homogeneous, and remained typical quasi-spherical as HBc VLPs.

To verify whether the CpG ODNs bound on Au NPs were essential for encapsulation, control experiments were also conducted. In the first control, the assembly that was conducted between naked Au NPs alone was mixed with HBc VLPs, as shown in Fig. 3b. Lots of empty HBc VLPs and plain Au NPs were observed in Fig. 3b. Another control experiment was also conducted, in which CpG ODNs with the absence of Au NP were incubated with HBc subunits. The encapsulation capacity of HBc VLPs with CpG was evaluated by agarose gel electrophoresis (Fig. 3c). It was observed that CpG ODNs encapsulated into HBc VLPs exhibited no mobility in the electromobility shift assay (lanes 2) while free CpG ODNs (lane 1) moved at its usual position. The result showed complete retardation of CpG ODNs through encapsulation, confirming that the formation of CpG@HBc VLPs was achieved. These results demonstrated that CpG ODNs played a key role to the encapsulation. Since the C-terminal sequence of full-length HBc VLPs contains 42 amino acids (aa 141–183) with four highly positive charged reduplicative arginine-rich blocks [32, 33], which were responsible for encapsulation of nucleic acids. The CpG-Au NPs were packaged inside the VLPs through binding to capsid-internal arginine repeats.

### Evaluation of Humoral Immune Response in Mice

**Fig. 4:**
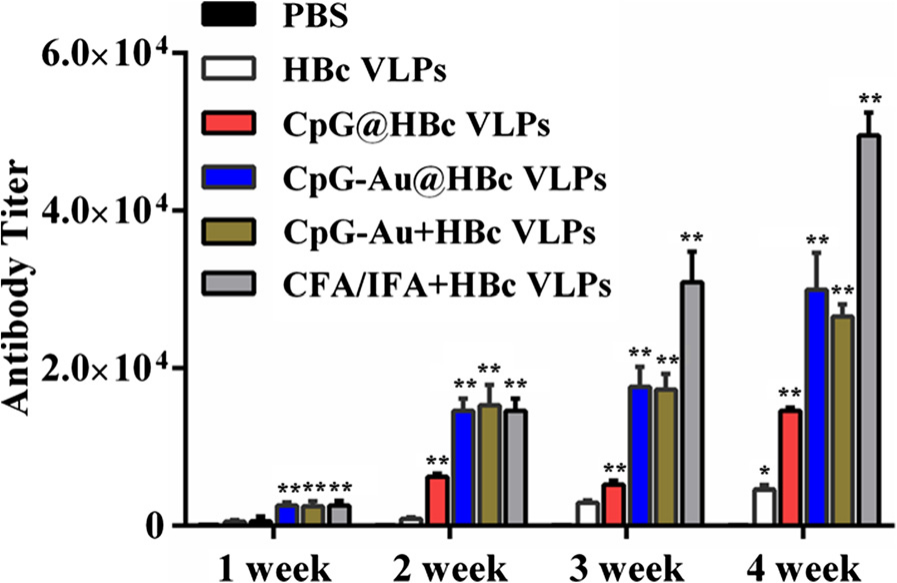
HBc-specific IgG antibody titers (reciprocal serum dilution) induced by HBc VLPs, CpG@HBc VLPs, CpG-Au@HBc VLPs, CpG-Au + HBc VLPs, CFA/IFA + HBc VLPs, and PBS in BALB/C mice, respectively. Mice (*n* = 6) were intraperitoneally vaccinated on days 0, 7, 14, and 21 as described in the “Methods” section and detected antibody titers at the end of each week. The results represent anti-HBc titers as the means from six mice ± standard deviation (SD). The statistical significance of the results was analyzed (PBS group served as control, all the other groups contrasted with PBS group) and indicated: *p < 0.05; **p < 0.01

To further evaluate the immunostimulatory properties of the CpG-Au@HBc VLPs nanocomposite, both humoral and cell-mediated immune response were considered. Intraperitoneal immunisations of BALB/c mice were performed with HBc VLPs, CpG-Au@HBc VLPs, CpG-Au + HBc VLPs, CpG@HBc VLPs, and HBc VLPs mixed with conventional Freund’s adjuvants (CFA/IFA + HBc VLPs), which were widely applied to induce both humoral and cellular immune response, respectively. Humoral immune responses were documented on days 7, 14, 21, and 28 by evaluating the anti-HBc titer in the sera of mice. As shown in Fig. 4, the significant differences of HBc titer among the five groups were increased after immunization for 4 weeks. Moreover, increasing the times of immunization was efficacious to increasing anti-HBc titer. Single HBc VLPs induced relatively low HBc-specific IgG antibody (1:4600) when comparing with the adjuvant immune groups (CpG-Au@HBc VLPs, CpG-Au + HBc VLPs, CpG@HBc VLPs, and CFA/IFA + HBc VLPs) after the fourth immunization. These results revealed the weak ability of HBc to stimulate strong humoral immune response when immune mice without any adjuvant. The CpG@HBc VLPs group, CpG-Au + HBc VLPs group, and CpG-Au@HBc VLPs group also showed humoral immune stimulation. The titer induced by CpG-Au@HBc VLPs group has no significant difference with CpG-Au + HBc VLPs group, while presented obviously improved antibody response than CpG@HBc VLPs group. After 4 weeks of immunization, the anti-HBc response induced by CpG-Au@HBc VLPs was 1:29,900, exceeding ~2.0 times of that induced by CpG@HBc VLPs (1:14,600). It is thus suggested that CpG-Au@HBc VLPs could enhance HBc-specific immune responses might due to Au NPs enhancing the property of CpG ODNs to stimulate humoral immune responses. However, the highest end point dilution titers of anti-HBc (1:49,500) was induced by CFA/IFA + HBc VLPs. This may due to the large doses of CFA (50 μg) and IFA (50 μg) used in mice immunization, which could cause greater side effects than CpG ODNs.

### Detection of CD8^+^ and CD4^+^ Cell Responses in Splenocytes

**Fig. 5:**
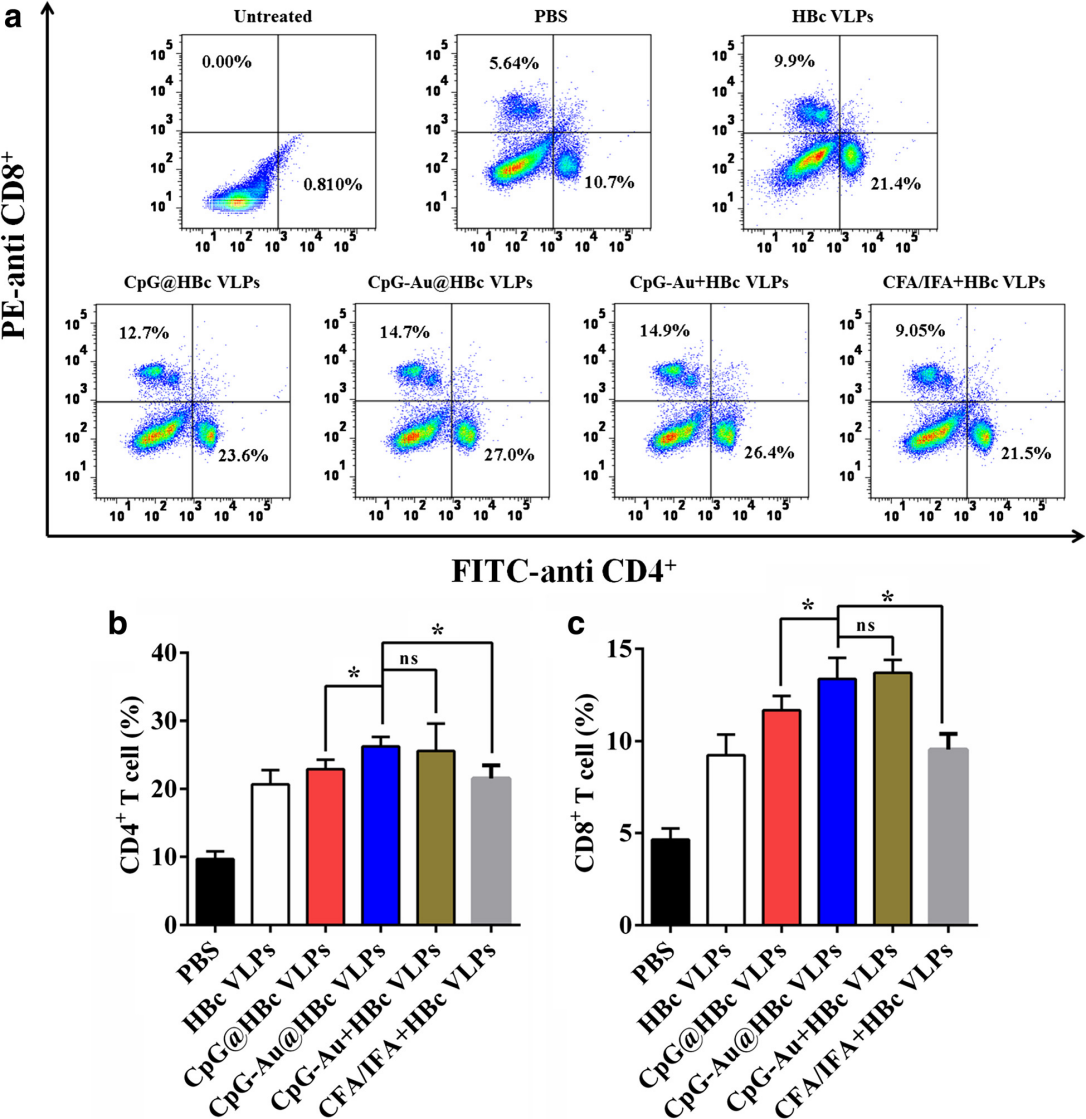
Induction of HBc-specific CD4^+^ and CD8^+^ T cells after immune with HBc VLPs, CpG@HBc VLPs, CpG-Au@HBc VLPs, CpG-Au + HBc VLPs, CFA/IFA + HBc VLPs, and PBS, respectively. The splenocyte surface molecules expressions were determined with flow cytometry analysis. A representative result of three independent experiments was shown in (**a**); the data shown in (**b**) and (**c**) were determined as means ± SD from three experiments. The statistical significance of the results was analyzed and indicated: *p < 0.05. *ns* no significant difference

T cells play key roles in cellular immune response [34]. The T cell response to HBc VLPs was determined using a number of FACS-based T cell assays to evaluate the induced CD4^+^ and CD8^+^ T cells in splenocytes, which were known as two prime T cell subsets. The CD4^+^ T cells are mainly T helper (Th) cells, and their responses can be divided into two types, T helper 1 (Th1) and T helper 2 (Th2), based upon cytokine secretion and effector function [35]. Strong cellular immunity associated with Th1-type immune response is thought to be essential for the control of intracellular pathogens. Whereas strong humoral immunity, which can be initiated and induced by Th2-type immune response, appears to be essential for the control of extracellular pathogens [36]. It was demonstrated that HBc protein possesses many Th cell epitopes and CTL epitopes [9]. These epitopes contributed to enhancing the immunogenicity of HBc to activate cellular immunity. Flow cytometry analysis in Fig. 5a indicated that the production of CD4^+^ T cell subset was significantly higher in mice immunized with HBc than those immunized with PBS (*p* < 0.05), suggesting that all of the vaccine formulations (HBc, CpG-Au@HBc VLPs, CpG-Au + HBc VLPs, CpG@HBc VLPs, and CFA/IFA + HBc VLPs) could activate CD4^+^ T cell subsets. The CD4^+^ T cell in mice immunized with CpG-Au@HBc VLPs (26.3 %) was much higher than those immunized with CpG@HBc VLPs (22.9 %; *p* < 0.05) and CFA/IFA + HBc VLPs (21.5 %; *p* < 0.05), while has no significant difference with CpG-Au + HBc VLPs (25.7 %; *p* > 0.05). The other T cell subset evaluated here was CD8^+^ T cells. The antigen-specific CD8^+^ T cells are mainly CTL, which are critical for protecting against intracellular pathogens. Flow cytometry analysis of CD8^+^ T cells in Fig. 5b gave the similar results to that of CD4^+^ T cells. The production of CD8^+^ T cell subsets were also significantly higher in mice immunized with HBc than those immunized with PBS (*p* < 0.05). The production of CD8^+^ T cells elicited by CpG-Au@HBc VLPs (13.4 %) revealed higher level than those induced by CpG@HBc VLPs (11.7 %; *p* < 0.05) and CFA/IFA + HBc VLPs (9.47 %; *p* < 0.05). Hence, CpG-Au@HBc VLPs had great potential as immunostimulatory vaccine formulation for the generation of specific T cell responses than CpG@HBc VLPs and CFA/IFA + HBc VLPs. Besides, Au NPs made the vaccine formulation much more efficient to induce immune response.

### IFN-γ, IL-2, and IL-4 Cytokine ELISA

**Fig. 6:**
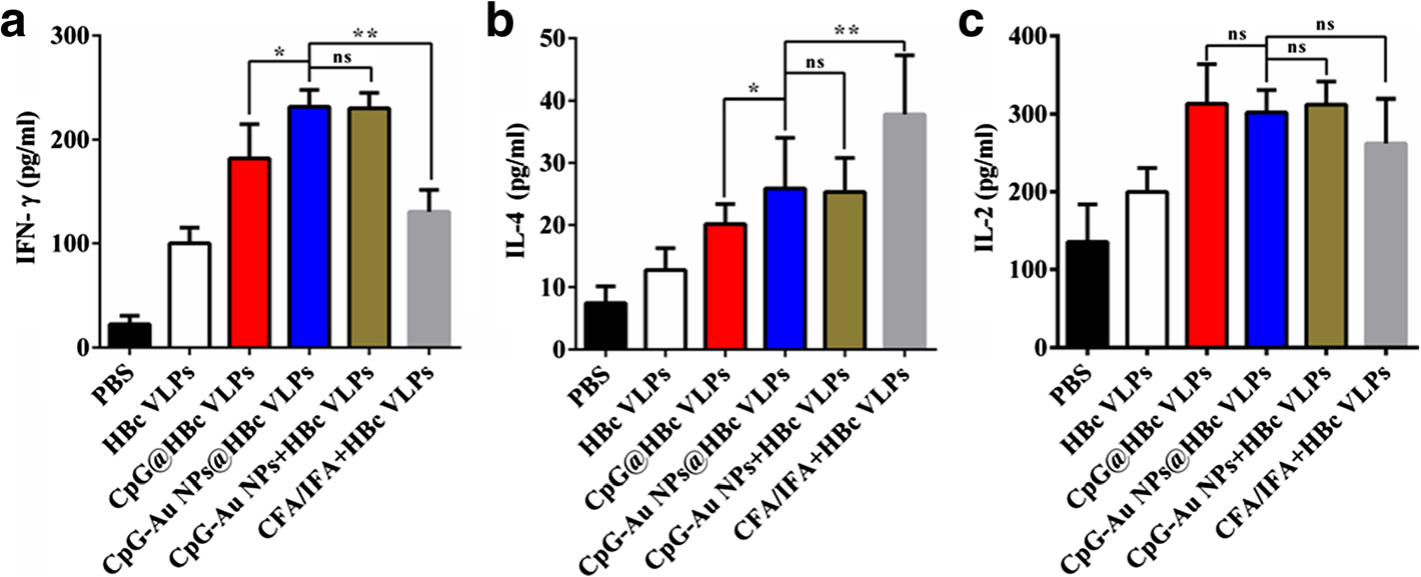
The secretion level of cytokines **a** IFN-γ, **b** IL-4, and **c** IL-2 in serum of BALB/c mice after immunization with HBc VLPs, CpG@HBc VLPs, CpG-Au@HBc VLPs, CpG-Au + HBc VLPs, CFA/IFA + HBc VLPs, and PBS, respectively. The data shown is a representative of the three independent experiments, and the statistical significance of the results was analyzed and indicated *p < 0.05; **p < 0.01. *ns* no significant difference

It is well known that cytokines IFN-γ can induce Th1 differentiation and IL-4 can induce Th2 differentiation. Another cytokines IL-2 can induce both Th1/2 differentiation [35]. The secretions of these cytokines were evaluated to study the effects of CpG-Au@HBc VLPs on both humoral and cellular immune responses. As shown in Fig. 6, immunization with CFA/IFA + HBc VLPs resulted in Th2-dominated immune responses characterized by HBc-specific secretion of IL-4 but not IFN-γ and IL-2. In contrast, immunization with CpG ODNs (CpG@HBc VLPs, CpG-Au + HBc VLPs, and CpG-Au@HBc VLPs groups) generated high levels of HBc-specific IFN-γ and IL-2 secretion and decreased HBc-specific IL-4 production. The result was in accordance with the previous study that CpG ODNs could switch the immune response to a Th1-dominated cytokine pattern [16]. The production of IL-4 triggered by CpG-Au@HBc VLPs was more than that triggered by CpG@HBc VLPs (*p* < 0.05), but is considerably less than that triggered by CFA/IFA + HBc VLPs (*p* < 0.01), and showed no significant difference with CpG-Au + HBc VLPs. This result was consistent with the result of anti-HBc titer in Fig. 4, demonstrated that CpG-Au@HBc VLPs could induce Th2 cell immune response to activate higher humoral immune response than CpG@HBc VLPs. On the other hand, the secretion of IFN-γ was significantly higher in BALB/c mice immunized with CpG-Au@HBc VLPs than other groups (*p* < 0.05). However, the secretion of cytokine IL-2 presented no significant difference (*p* > 0.05) among CpG@HBc VLPs, CpG-Au + HBc VLPs, CpG-Au@HBc VLPs, and CFA/IFA + HBc VLPs. It was thus indicated that strong Th1 cell immune response was also stimulated by CpG-Au@HBc VLPs.

In general, the above results suggested that CpG-Au@HBc VLPs could induce stronger cellular immune response when compared with CpG@HBc VLPs and CFA/IFA + HBc VLPs, respectively. Meanwhile, this nanocomposite could also stimulate greater humoral immune response when contrast with CpG@HBc VLPs. Such robust immune responses caused by low dosage of adjuvant (CpG ODNs) could help to reduce the side effects in mice. Besides, Au NPs of this nanocomposite played an important role in inducing both Th1- and Th2-dominated response.

## Conclusions

In summary, a highly monodisperse and uniform CpG-Au@HBc VLPs nanocomposite was developed through a dissociation and reassembling process. The obtained adjuvant-containing VLPs exhibited both strong humoral and cellular immune stimulation ability. Noteworthy, Au NPs, which were conjugated with CpG ODNs, could enhance the immunogenicity of the nanocomposite through helping to stimulate both Th1- and Th2-dominated immune response. This new type of immunostimulatory nanocomposites is thus expected to be as a potential vaccine for prophylactic and therapeutic application, even for tumor immunotherapy.

## Abbreviations

Au NPs, gold nanoparticles; Au NRs, gold nanorods; CFA, complete Freund’s adjuvant; CFA/IFA-HBc VLPs, HBc VLPs mixed with conventional Freund’s adjuvants; CpG ODNs, oligodeoxynucleotides-containing un-methylated CpG motifs; CpG@HBc VLPs, HBc VLPs encapsulated CpG ODNs; CpG-Au@HBc VLPs, HBc VLPs encapsulated CpG-Au conjugates; CTL, cytotoxic T lymphocyte; *E. coli*, *Escherichia coli*; HBc VLPs, hepatitis B core protein virus-like particles; HBcAg, HBc antigen; IFA, incomplete Freund’s adjuvant; IFN-γ, interferon-gamma; IL-2, interleukin-2; IL-4, interleukin-4; NK cells, natural killer cells; Th, T helper
